# Acute Effects of Exercise on Blood Pressure: A Meta-Analytic Investigation

**DOI:** 10.5935/abc.20160064

**Published:** 2016-05

**Authors:** Elizabeth Carpio-Rivera, José Moncada-Jiménez, Walter Salazar-Rojas, Andrea Solera-Herrera

**Affiliations:** 1 School of Physical Education and Sports, University of Costa Rica - Costa Rica; 2 Human Movement Sciences Research Center (CIMOHU), University of Costa Rica - Costa Rica

**Keywords:** Blood pressure, Meta-analysis, Physical activity, Post-exercise hypotension, Training, Acute effect

## Abstract

Hypertension affects 25% of the world's population and is considered a risk factor for cardiovascular disorders and other diseases. The aim of this study was to examine the evidence regarding the acute effect of exercise on blood pressure (BP) using meta-analytic measures. Sixty-five studies were compared using effect sizes (ES), and heterogeneity and Z tests to determine whether the ES were different from zero. The mean corrected global ES for exercise conditions were -0.56 (-4.80 mmHg) for systolic BP (sBP) and -0.44 (-3.19 mmHg) for diastolic BP (dBP; z ≠ 0 for all; p *<* 0.05). The reduction in BP was significant regardless of the participant's initial BP level, gender, physical activity level, antihypertensive drug intake, type of BP measurement, time of day in which the BP was measured, type of exercise performed, and exercise training program (p *<* 0.05 for all). ANOVA tests revealed that BP reductions were greater if participants were males, not receiving antihypertensive medication, physically active, and if the exercise performed was jogging. A significant inverse correlation was found between age and BP ES, body mass index (BMI) and sBP ES, duration of the exercise's session and sBP ES, and between the number of sets performed in the resistance exercise program and sBP ES (p *<* 0.05). Regardless of the characteristics of the participants and exercise, there was a reduction in BP in the hours following an exercise session. However, the hypotensive effect was greater when the exercise was performed as a preventive strategy in those physically active and without antihypertensive medication.

## Introduction

Exercise training has been shown to reduce blood pressure (BP).^1-9^ However, studies reporting a reduction in BP resulting from chronic exercise might disregard an acute effect following the exercise session (*i.e.*, post-exercise hypotension [PEH]) that is lost over time.^4^ Although the mean reductions in ambulatory systolic BP (sBP) and diastolic BP (dBP) monitoring over 24 hours are 3.2 mmHg and 1.8 mm Hg, respectively,^10^ the magnitude of the reduction is greater during the first few hours after the exercise, to the point that some subjects with hypertension achieve normal BP values.

The PEH response is measured by comparing BP values after an exercise with the values in a control day in which the exercise is not performed, or by comparing BP values before and after an exercise session.^5^ However, findings in the literature are contradictory, not only regarding the conclusion of whether an acute exercise elicits a reduction in BP, but also about the magnitude and duration of the PEH response. These contradictions may be partially explained by the characteristics of the samples (*i.e.*, hypertensives *versus* normotensives),^10-18^ use of antihypertensive medication,^16,17^ training status,^19-23^ participants' age,^24-27^ and characteristics of the measurement performed. This relates to whether the BP was measured at rest or by ambulatory monitoring,^5^ since the latter is more effective in distinguishing the "white coat syndrome" (a transient elevation in BP when the measurements are performed in a laboratory or in the clinic).^28,29^ Finally, other confounding factors include the duration of the measurement^5^ and characteristics of the exercise, such as type (*i.e.*, aerobic or resistance),^30,31^ intensity,^8,32-35^ duration of the session,^7,36,37^ muscles involved,^7^ whether the exercise is performed intermittently or continuously,^38^ and the time of day when it is performed.^39,40^

Given this plethora of ambivalent variables, the purpose of this meta-analysis was to determine the effect of acute exercise on the BP response and examine the role of moderator variables.

## Methods

*Search strategy*. A systematic search was conducted from August 8, 2012, to March 9, 2013, on the databases MEDLINE (Ovid), SciELO, SPORTDiscus, Google Scholar, ProQuest, SpringerLink, and PubMed. The following keywords were used alone and in combination: "acute effect of exercise", "blood pressure", "hypertension", "post-exercise hypotension", and "physical activity". We performed a hand search of the reference lists of the retrieved studies to detect manuscripts not found by the search in the electronic engines mentioned above.

*Inclusion criteria*. Studies were included in this meta-analysis if they: 1) were published in English, 2) reported the effect of exercise on BP in the minutes or hours following the training session, 3) reported the mean and standard deviation (SD) or standard error values of the BP in the experimental and control groups before and after the exercise, 4) included only humans, and 5) performed BP readings at rest or ambulatory measurements in the hours that followed the exercise session.

*Exclusion criteria*. Studies were excluded from this meta-analysis if their data: 1) were used to publish other manuscripts, to prevent their results from being included more than once in our database (*i.e.*, studies using the same dataset were taken into consideration only once), and 2) resulted from an interaction between exercise and medication or intervention to evaluate possible physiological mechanisms that might explain the occurrence of PEH.

*Variable coding*. The coded moderator variables included the characteristics related to the following: 1) studies (number of participants, study quality, experimental condition or group); 2) participants (BP level, gender, medication status, age, body mass index [BMI], physical activity level, maximum oxygen uptake [VO_2_max]); 3) BP measurement (type, duration and time of day when it was performed); and 4) exercise (type, training protocol, training mode, intensity, rest between sets or intervals, and number of exercises, sets, and repetitions). The quality of the studies was determined using the Jadad scale,^41^ in which the quality according to the total score is categorized as low when < 3 points, moderate when 3 points, and high when > 3 points. Multiple effect sizes (ES) for the same study were computed for trials with a repeated measures design including multiple interventions. Likewise, the ES was computed for the intervention or control groups when the information was available.

*Statistical analysis*. The following analyses were computed for each dependent variable (sBP and dBP). To calculate the ES, we followed the procedures described elsewhere.^42,43^ First, the ES was computed separately for the experimental and control conditions with the following formula:^43,44^*ES = (Mean_post-test_ - Mean_pre-test_) / SD_pre-test_*. Second, the ES was corrected taking into consideration the sample size using the following formula:^44^*ES_corrected_ = ES x 1 - (3 / 4 x m - 9)*. Once the global corrected ES was obtained, we determined the possibility of a "file-drawer effect" using the following formula:^45^*K_0_ = K (d_1_ - d_2_) / d_2_*; where K_0_ is the number of studies theoretically required to reduce the computed global ES to a non-significant ES, *K* is the number of meta-analyzed studies, *d_1_* is the global ES, and *d_2_* is the non-significant global ES, in this case, 0.20.^46^ The Z test was used to determine whether the ES were significantly different from zero.^43^ Statistical heterogeneity among the studies was assessed using Cochran's Q test, and the I^2^ index.^42^ One-way ANOVA was used to determine the global experimental ES and ES differences in the control conditions.^44^ One-way ANOVA for independent groups and Pearson's correlation were computed on the nominal and continuous moderator variables, respectively, when heterogeneity was found in the global ES. Tukey's *post-hoc* analyses were computed when significant *F* ratios were obtained. Analyses were performed using the software SPSS, version 20.0 (IBM Corporation, New York, USA). Significance was set *a priori* at p *<* 0.05.

## Results

**Chart 1:**
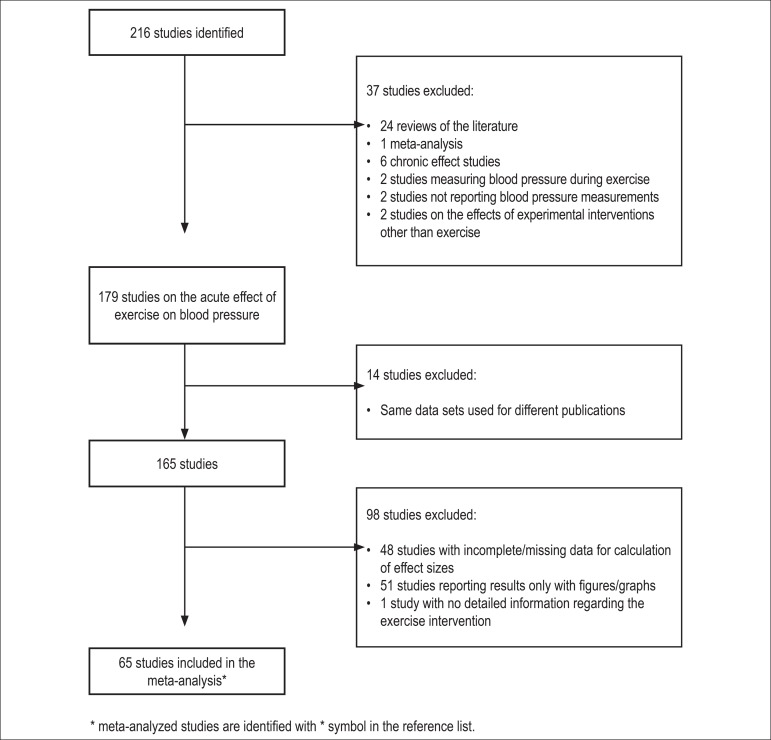
Study selection flow diagram.

Sixty-five studies (denoted by * in the reference list) out of 216 initial citations were included in the meta-analysis (Chart 1). The studies enrolled 1408 participants (931 males, 455 females, 22 with undisclosed gender), with a mean age of 36.1 ± 15.1 years, BMI of 25.9 ± 2.6 kg/m^2^ and VO_2_max of 33.1 ± 10.2 mL x min^-1^ x kg^-1^. Of these participants, 466 engaged in studies with a repeated measures design including experimental and control conditions; 309 participated in studies with a repeated measures design including only experimental conditions; 429 participated in studies with an independent measures design including only experimental groups; 204 participated in studies with an independent measures design in which 117 exercised; and 87 were controls. From this sample, 1101 ES were computed.

**Figure 1:**
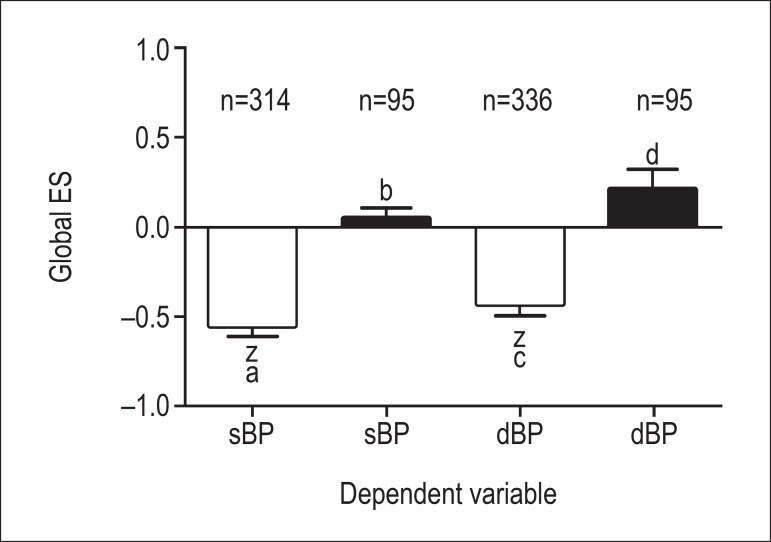
Global effect size of systolic and diastolic blood pressure. ES: effect size; sBP: systolic blood pressure; dBP: diastolic blood pressure; z: ES ≠ 0, p < 0.05; p < 0.05 between a and b, c and d. Open bars represent the experimental condition, and black bars represent the control condition.

All the obtained ES were included in the subsequent analysis given the lack of statistically significant differences in the quality of the moderator variable of the study for sBP (*F =* 1.91, p *=* 0.11) and dBP (*F =* 0.40, p *=* 0.81). Table 1 shows that, in contrast to the experimental condition, the corrected ES in the control condition were not different from zero. However, Cochran's Q test indicated that data from both experimental and control conditions were heterogeneous. Figure 1 shows the overall corrected ES for the experimental and control conditions for the dependent variables sBP and dBP. One-way ANOVA showed significant differences between control and experimental conditions regarding sBP and dBP (p *≤* 0.01 for all). Assessment of a file drawer effect determined that for global effects to be no longer significant, 122 significant unpublished studies were needed for sBP and 165 studies for dBP. In the control condition, while the Z score showed ES = 0, the Cochran's Q test found heterogeneity explained by the sBP (*F =* 13.90) and dBP (*F =* 5.37). Further analysis showed that the BP increased when measured later on during the day (p *≤* 0.01 for both). The experimental conditions not only showed heterogeneity in the obtained ES but also global ES ≠ 0 in sBP and dBP (Table 1).

**Table 1:** Global corrected ES, Z scores, Q statistic and I2 index heterogeneity tests, and post-session blood pressure change (Δ mmHg)

Experimental condition or group	Variable	ES ± SD	*Z*	*Q*	*I^2^*	*Δ* (mmHg)
Control	sBP	0.05 ± 0.56	-0.13	186.87*	95.18	0.53
	dBP	0.21 ± 1.10	1.81	329.84*	97.27	0.26
Experimental	sBP	-0.56 ± 0.90	-20.21^z^	1452.57*	99.38	-4.80
	dBP	-0.44 ± 1.14	-15.91^z^	751.47*	98.80	-3.19

ES: effect size; SD: standard deviation; Z: Z score; Q: Cochran Q test; I2: heterogeneity percentage; Δ: post-test minus pre-test change in blood pressure; sBP: systolic blood pressure; dBP: diastolic blood pressure; Z: Z score ≠ 0, p < 0.05;

* heterogeneous values, p < 0.05.

The results of the experimental condition on the two dependent variables are presented next.

**Figure 2:**
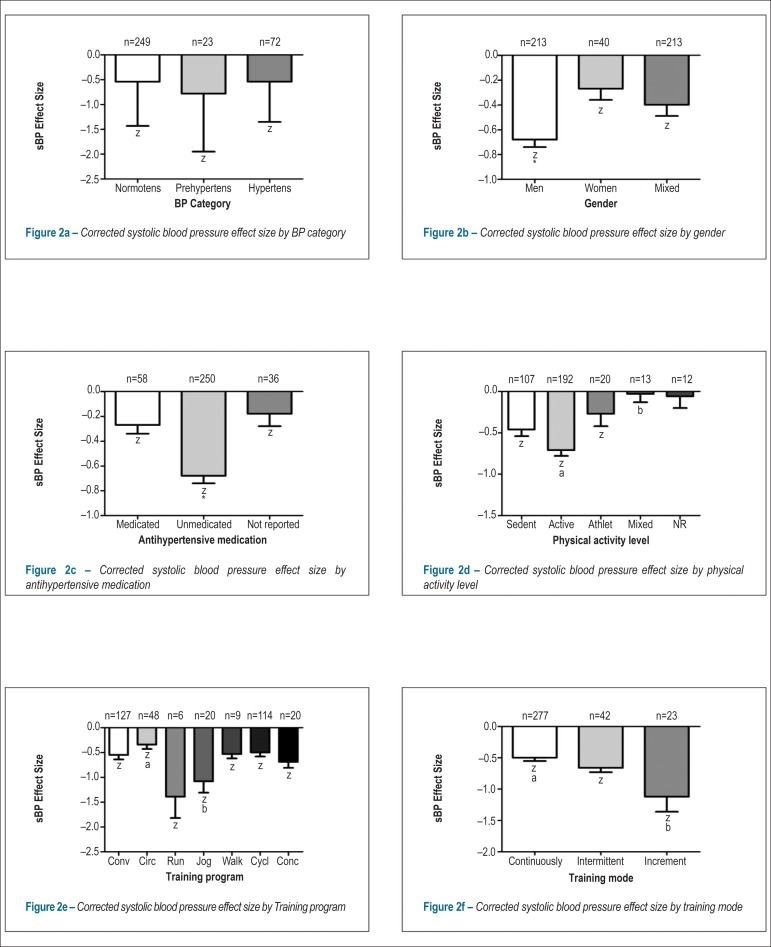
Corrected systolic blood pressure effect size by categorical variables. Normotens.: normotensive; Prehypertens.: prehypertensive; Hypertens.: hypertensive; BP: blood pressure; sBP: systolic blood pressure; z: ES ≠ 0, p < 0.05; *: different from others, p < 0.05; a and b: different between each other, p < 0.05; Conv.: Conventional resistance training; Circ.: Circuit resistance training; Run: running; Jog: jogging; Walk: walking; Cycl.: bicycling; Conc.: Concurrent training.

**Figure 3:**
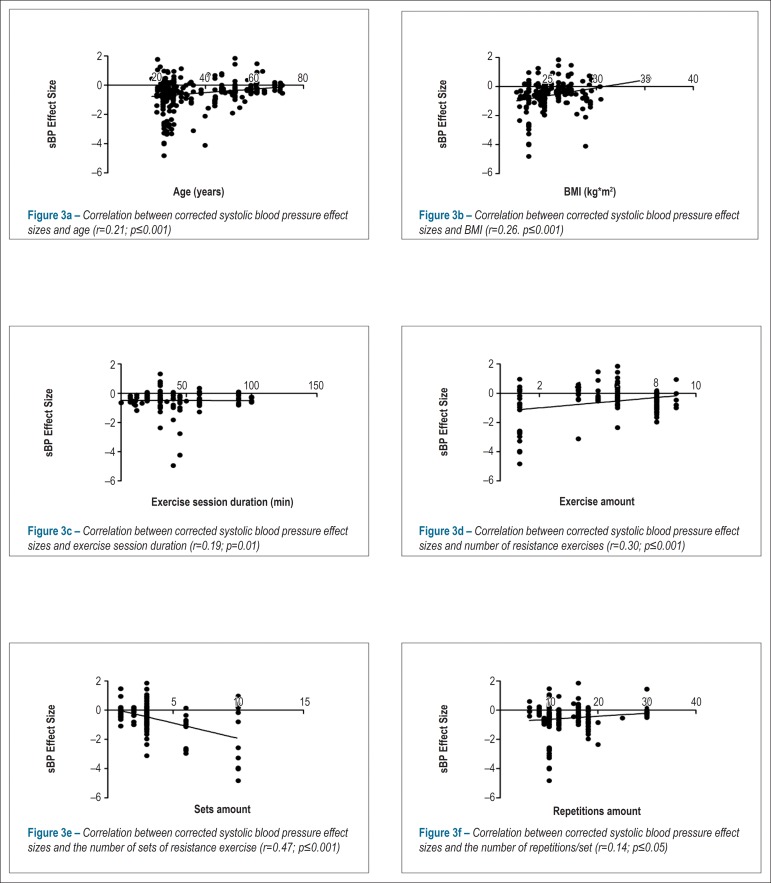
Correlation between corrected systolic blood pressure (sBP), effect sizes, and continuous variables. Note: sBP: systolic blood pressure; BMI: body mass index.

*Systolic Blood Pressure.* Table 2 shows the corrected mean sBP ES at different levels of the moderator variables. Results regarding the characteristics of the sample showed a significant decrease in sBP regardless of the initial BP levels, gender, antihypertensive drug intake, and physical activity level. However, *post-hoc* analyses detected a significantly larger ES in males (*F =* 5.58, p *=* 0.001, Figure 2b), and non-medicated (*F =* 8.76, p *=* 0.001, Figure 2c) and physically active subjects (*F =* 4.42, p *=* 0.002, Figure 2d). Results regarding the exercise characteristics showed that the sBP decreased significantly regardless of the exercise modality. Results were consistent for aerobic exercises such as running, jogging, walking, cycling, or a combination of these, as well as for conventional or circuit resistance training exercise. Nevertheless, reductions in sBP were significantly greater for jogging exercise compared with circuit resistance training exercise (*F =* 2.73, p *<* 0.01, Figure 2e). Significant sBP reductions were also found regardless of whether the exercise was performed continuously, intermittently, or increasingly. However, largest reductions occurred when the intensity increased during the exercise session (*F =* 5.50, p *=* 0.004, Figure 2f). Significant correlations were found for sBP (Table 3). Because in most cases the post-exercise BP decreased, the ES were negative, and therefore, the direction (*i.e.*, sign) of the correlations opposed to those commonly reported. For example, the higher the age of the participants, the lower the decrease in sBP (*r =* 0.21, p *=* 0.001, Figure 3a, Table 3). In addition, higher BMI values were associated with a lower decrease in sBP (*r =* 0.26, p *=* 0.001, Figure 3b). Also, the longer the duration of the exercise session the greater the reduction in sBP (*r =* -0.19, p *=* 0.01, Figure 3c), and the lower number of resistance exercises performed, the higher the decrease in sBP (*r =* 0.21, p *=* 0.001, Figure 3d). Finally, the greater the number of sets of resistance exercises, the greater the reduction in sBP (*r =* -0.47, p *=* 0.001, Figure 3e).

**Table 2:** Mean corrected sBP ES, Z scores, F-ratio, significance level, and post-exercise score change by moderator variable in the experimental group

Moderator variable	Coding scheme	*n*	Mean corrected ES ± SD	*Z*	*F*	p* ≤*	*Δ* (mmHg)
Sample characteristics	**BP category**				0.74	0.48	
	•Normotensive	249	-0.54 ± 0.89	-15.5*			-3.75
	•Prehypertensive	23	-0.78 ± 1.17	-4.4*			-5.80
	•Hypertensive	72	-0.54 ± 0.81	-13.1*			-8.13
	**Gender**				5.58	0.004	
	•Males	213	-0.68 ± 0.94	-20.5*			-4.95
	•Females	40	-0.27 ± 0.60	-4.44*			-3.98
	•Mixed	91	-0.40 ± 0.84	-6.95*			-4.81
	**Medication**				8.76	0.001	
	•Medicated •Nonmedicated •Unreported	58 250 36	-0.27 ± 0.50 -0.68 ± 0.97 -0.18 ± 0.57	-6.19* -19.4* -2.71*			-4.90 -5.08 -2.74
	**Physical activity level**				4.42	0.002	
	•Sedentary	107	-0.46 ± 0.79	-11.2*			-5.05
	•Active	192	-0.71 ± 0.98	-19.9*			-5.45
	•Athletes	20	-0.27 ± 0.66	-2.58*			-1.64
	•Mixed	13	-0.03 ± 0.36	0.35			-0.75
	•Unreported	12	-0.06 ± 0.47	-1.02			-1.89
Measurement features	**Type of measurement**				0.55	0.46	
	•Resting	306	-0.56 ± 0.92	-18.1*			-4.81
	•Ambulatory	40	-0.46 ± 0.66	-8.71*			-4.31
	**Time of day**				2.20	0.11	
	•Morning	101	-0.71 ± 1.16	-17.6*			-4.58
	•Afternoon	9	-0.74 ± 1.05	-4.9*			-5.11
	•Unreported	234	-0.49 ± 0.74	-11.7*			-4.89
Exercise characteristics	**Exercise type**				0.97	0.38	
	•Aerobic	148	-0.62 ± 0.87	-16.1*			-6.22
	•Resistance training	175	-0.49 ± 0.95	-11.5*			-3.36
	•Concurrent	20	-0.69 ± 0.52	-7.7*			-7.33
	**Training program**				2.73	0.01	
	•Conventional (RT)	127	-0.55 ± 1.04	-10.4*			-3.24
	•Circuit (RT)	48	-0.34 ± 0.64	-4.99*			-3.7
	•Running (AT)	6	-1.39 ± 1.05	-6.16*			-8.53
	•Jogging (AT)	20	-1.08 ± 1.02	-8.86*			-8.7
	•Walking (AT)	9	-0.53 ± 0.27	-6.52*			-7.81
	•Bicycling (AT)	114	-0.50 ± 0.82	-11.2*			-5.45
	•Mixed	20	-0.69 ± 0.52	-7.7*			-7.33
	**Mode (RT, AT)**				5.50	0.004	
	•Constant	277	-0.50 ± 0.91	-16.5*			-4.00
	•Intermittent	42	-0.67 ± 0.44	-10.7*			-7.12
	•Incremental	23	-1.12 ± 1.16	-8.03*			-10.87
	**Rest/series (RT)**				0.24	0.87	
	•12 min	163	-0.54 ± 0.96	-12.6*			-3.86
	•35 min	22	-0.52 ± 0.65	-4.14*			-5.09
	•Unreported	12	-0.45 ± 0.66	-3.66*			-4.75

BP: blood pressure; sBP: systolic blood pressure; ES: effect size; RT: resistance training; AT: aerobic training; Mode: both AT and RT are included;

* Z score ≠ 0, p < 0.05.

**Table 3:** Pearson’s correlation of mean sBP and dBP, corrected ES, and moderator variables according to the coding scheme

Characteristics of the moderator variable	Coding scheme	BP	r =	p ≤
Participants	•Age	sBP	0.21	0.001
		dBP	0.12	0.03
	•Weight	sBP	0.007	0.24
		dBP	-0.06	0.37
	•Body mass index	sBP	0.26	0.001
		dBP	0.09	0.14
	•VO2max	sBP	-0.03	0.70
		dBP	-0.04	0.61
Measurement	•Measurement duration	sBP	0.08	0.15
		dBP	-0.07	0.21
Exercise	•Exercise intensity estimated from the VO_2_max	sBP	-0.16	0.11
		dBP	0.04	0.72
	•Exercise intensity estimated from the HRR	sBP	0.11	0.56
		dBP	-0.10	0.57
	•Exercise intensity estimated from the HRmax	sBP	-0.19	0.58
		dBP	-0.47	0.14
	•Exercise intensity estimated from the anaerobic threshold	sBP	0.33	0.17
		dBP	0.35	0.15
	•Exercise intensity estimated from 1RM	sBP	-0.05	0.51
		dBP	-0.04	0.58
	•Duration of the exercise session	sBP	-0.19	0.01
		dBP	-0.08	0.32
	•Number of RT exercises	sBP	0.30	0.001
		dBP	-0.20	0.006
	•Number of sets	sBP	-0.47	0.001
		dBP	-0.02	0.75
	•Number of repetitions	sBP	0.14	0.05
		dBP	0.07	0.37

VO_2_max: maximal oxygen consumption; HRR: heart rate reserve; HRmax: maximal heart rate; 1RM: one repetition maximum; RT: resistance training; BP: blood pressure; sBP: systolic blood pressure; dBP: diastolic blood pressure.

*Diastolic Blood Pressure.* Table 4 shows the corrected mean dBP ES at different levels of the moderator variables. Results regarding the characteristics of the subjects showed a significant decrease in dBP regardless of the initial BP level, gender, antihypertensive drug intake, and physical activity level. However, *post-hoc* analyses detected a significantly larger ES in non-medicated samples (*F =* 4.26, p *<* 0.02). This finding is consistent with the sBP response depicted in Figure 2c. Results regarding the exercise characteristics showed that the dBP decreased significantly regardless of the exercise modality. Most of the results were consistent for aerobic exercises such as jogging, cycling, and a combination of these, as well as for conventional or circuit resistance training exercise. However, as depicted in Table 4, the largest reductions in dBP occurred when jogging was the exercise mode (*F =* 4.09, p *<* 0.001). Interestingly, dBP ES were not different from zero when the participants walked. Significant correlations were found for dBP (Table 4). Also, the higher the age of the participants, the lower the reduction in dBP (*r =* 0.12, p *=* 0.03), and the greater the number of resistance exercises performed, the higher the decrease in dBP (*r =* -0.20, p *=* 0.006).

**Table 4:** Mean corrected dBP ES, Z score, F ratio, significance level, and post-exercise score change by moderator variable in the experimental group

Characteristics of the moderator variable	Coding scheme	*n*	Mean corrected ES ± SD	*Z*	*F*	p* ≤*	Δ (mmHg)
Sample	**BP category**				1.8	0.17	
	•Normotensive	249	-0.44 ± 0.97	-13.9*			-3.07
	•Prehypertensive	20	-0.85 ± 3,16	-4.08*			-5.28
	•Hypertensive	67	-0.30 ± 0.44	-6.72*			-3.02
	**Gender**				0.41	0.67	
	•Male	207	-0.48 ± 1.38	-12.2*			-3.4
	•Female	40	-0.34 ± 0.59	-4.75*			-2.85
	•Mixed	89	-0.38 ± 0.61	-9.20*			-2.85
	**Medication**				4.26	0.02	
	•Medicated	58	-0.20 ± 0.43	-4.54*			-1.79
	•Nonmedicated	242	-0.55 ± 1.31	-15.7*			-3.87
	•Unreported	36	-0.08 ± 0.38	-1.04			-0.88
	**Physical activity level**				0.87	0.49	
	•Sedentary	105	-0.48 ± 1.49	-8.09*			-3.25
	•Active	186	-0.47 ± 1.03	-12.9*			-3.49
	•Athletes	20	-0.35 ± 0.32	-3.70*			-2.72
	•Mixed	13	-0.25 ± 0.36	-4.46*			-2.36
	•Unreported	12	-0.10 ± 0.63	0.14			0.22
BP measurement	**Type of measurement**				1.47	0.23	
	•Resting	296	-0.47 ± 1.21	-15.3*			-3.36
	•Ambulatory	40	-0.23 ± 0.37	-4.64*			-1.92
	**Time of day**				1.03	0.36	
	•Morning	99	-0.31 ± 0.55	-10.5*			-1.97
	•Afternoon	9	-0.29 ± 0.68	-1.89			-1.33
	•Unreported	228	-0.50 ± 1.33	-11.91			-3.79
Exercise	**Exercise type**				0.81	0.45	
	•Aerobic	141	-0.53 ± 1.61	-10.2*			-3.80
	•Resistance training	175	-0.38 ± 0.64	-11.4*			-2.73
	•Concurrent	20	-0.29 ± 0.34	-4.51*			-2.93
	**Training program**				4.09	0.001	
	•Conventional (RT)	127	-0.43 ± 0.67	-10.8*			-2.84
	•Circuit (RT)	48	-0.27 ± 0.54	-3.77*			-2.43
	•Running (AT)	6	-0.77 ± 0.99	-4.00*			-3.90
	•Jogging (AT)	18	-1.66 ± 3.20	-7.80*			-10.83
	•Walking (AT)	7	-0.19 ± 0.49	-0.45			-0.84
	•Bicycling (AT)	107	-0.36 ± 1.20	-6.79*			-2.82
	•Mixed	20	-0.29 ± 0.34	-4.51*			-2.93
	**Mode (RT, AT)**				0.44	0.64	
	•Constant	277	-0.46 ± 1.24	-14.1*			-3.24
	•Intermittent	39	-0.28 ± 0.30	-4.63*			-2.55
	•Incremental	17	-0.47 ± 0.56	-6.07*			-4.29
	**Rest/series (RT)**				0.54	0.66	
	•12 min	163	-0.35 ± 0.58	-11.1*			-2.67
	•35 min	20	-0.39 ± 0.67	-2.83*			-3.14
	•Unreported	11	-0.54 ± 0.74	-4.35*			-2.65

BP: blood pressure; dBP: diastolic blood pressure; ES: efect size; RT: resistance training; AT: aerobic training; Mode: both, AT and RT are included;

* Z score ≠ 0, p < 0.05

## Discussion

The purpose of this meta-analysis was to determine the effectiveness of acute exercise interventions on the BP response. Although initially we intended to find the intensity, duration, and type of exercise that best reduced BP, we found that regardless of the participant, measurement features, and exercise characteristics, there was a reduction in BP in the hours that followed an exercise session. The reductions in BP following an exercise session were demonstrated by the corrected ES significantly different from zero in the experimental conditions. Significant ES were found for sBP (-0.56 or -4.8 mm Hg) and dBP (-0.44 or -3.2 mm Hg). The ES for the controls conditions were equal to zero.

The magnitude of the ES is considered moderate when between 0.41 and 0.70.^46^ From a clinical perspective, epidemiological studies indicate that a decrease of 2 mmHg in the sBP is likely to reduce the mortality associated with stroke by 6% and coronary heart disease by 4%, whereas a reduction of 5 mmHg is likely to reduce the risk of these diseases by 14% and 9%, respectively.^1,47^ Therefore, the reductions of 3 to 4 mmHg found in this meta-analysis confirm the importance of acute exercise as a non-pharmacological treatment of hypertension.

The fact that the ES in the control condition was not different from zero indicates that there was no contamination by extraneous variables in this set of studies. The heterogeneity of the data from the control condition might have been partially explained by the significant differences between measurements taken in the afternoon as opposed to the morning. This finding suggests a confounding effect of the circadian rhythm in hemodynamic variables, given the reductions in BP, heart rate, cardiac output, and stroke volume as the night approaches.^48^ Other aspects may also influence this response, for instance, the fact that the BP measurement in the control condition was affected by exercise performed in the previous 48 hours.^49^ Therefore, both factors must be considered in the design of future research protocols.

In the case of the corrected ES arising from the experimental condition, it is noteworthy that although all participants benefited from exercise to lower the sBP, males achieved greater reductions than females. This finding is consistent with those of other studies^50^ that have suggested that females have a lower support of the autonomic tone necessary to regulate BP, as well as a lower effectiveness of the components that regulate the baroreflex. However, the same authors reported as a limitation of the study a failure to standardize the time of the menstrual cycle in the group of studied females. Evidence suggests that the different phases of the menstrual cycle are involved in the regulation of the autonomic nervous system.^51^ While we computed 213 ES for males, we computed only 40 ES for females. Researchers have apparently neglected the female population, probably due to a fear that the menstrual cycle might confound the findings due to its involvement in BP regulation. Although the PEH can be reached at any point during the menstrual cycle in normotensive women, it is greater if the woman exercises during the early follicular phase.^52^ However, further investigation is required on this topic to determine potential physiological mechanisms responsible for PEH, for instance, whether an interaction exists between gender, age, and arterial stiffness.^53^

Based on speculations from previous findings,^10^ we expected to find a greater PEH in hypertensive subjects than in prehypertensive and normotensive ones. However, the level of the participants' BP had no influence on the findings of the present study. This difference might be explained by the inclusion of non-medicated hypertensive and normotensive subjects in the study by Pescatello and Kulikowich;^10^ therefore, given a higher initial BP there was also a greater change in post-exercise BP when determined by ambulatory measurement. Although the PEH was significant in normotensive, prehypertensive, and hypertensive patients in the present study, there were no differences between these categories. Moreover, there were significantly greater changes in non-medicated participants compared with medicated ones. This finding might be explained by the interaction between medication intake and exercise intervention.^5^ Another feasible explanation for our findings opposing those by others^10^ might have been that some participants were classified as "medicated hypertensive", and therefore, BP values were close to or within the normal range. If this explanation holds true, the "baseline" law^8,10,54^ also seems to apply in the present study. In other words, since BP values were close to normal even in hypertensive subjects (*i.e.*, baseline), it is harder to achieve a lower BP following an exercise session. Therefore, these speculations deserve to be investigated with further post-meta-analytical studies, since the physiological mechanisms potentially explaining these findings are largely unknown.

Physically active individuals achieved higher BP decreases after the exercise session. This was observed even though the PEH occurred independently from the level of physical activity of the participants. This seems to support the theory proposed by some authors^55^ who observed that some physiological mechanisms that chronically reduce BP also play a role in the onset of PEH. For example, exercise training has been shown to cause a systemic adaptation of the arterial wall in healthy individuals,^56^ which might translate to better arterial vessel compliance that may facilitate the decrease in peripheral resistance following an exercise session.

We observed in this study an inverse association between age and PEH. Increasing age decreases the magnitude of PEH. As a person ages, there is an increase in arterial stiffness that results from progressive destruction of the elastic fibers, a decrease in capillary density, and an increase in arteriolar wall thickness. These structural and functional changes, in turn, increase vascular resistance and limit the response to vasodilator agents released during exercise.^57^ Similarly, if the VO_2_max is greatest when the person is young and active, then the relationship between a higher VO_2_max and a greater decrease in sBP could also be explained by the aforementioned physiological mechanisms.

The finding that a lower BMI was associated with a greater reduction in sBP is in line with evidence showing that adipose tissue accumulation, especially in the abdominal area, is linked to several mechanisms leading to hypertension, including sympathetic overactivity, endothelial dysfunction, arterial stiffness, and inflammation.^55,58,59^ The implications of these findings are significant, given that a large proportion of the world population is hypertensive and obese; therefore, maintaining a normal BMI could lead in many cases to a greater hypotensive effect following an exercise session.^60^

More than a decade ago, the American College of Sports Medicine (ACSM),^3^ recommended that resistance exercise should be accompanied by aerobic exercise. Recent studies attempted to determine whether resistance exercise alone could produce the same hypotensive effect than aerobic exercise.^31,61-62^ Motivated by the increase in the number of these studies, we decided to meta-analyze the type of exercise as a moderator variable. We found that both aerobic and resistance exercises alone were able to induce a hypotensive effect.

In this study, we found jogging to be the exercise modality that elicits the greater magnitude of sBP and dBP changes. Other findings were that walking does not reduce the dBP; that the longer the duration of the exercise session, the greater the sBP reduction; and that incremental exercise protocols produced the highest reductions in sBP. These findings seem to agree with a previous report^63^ that associated the PEH with the total exercise workload and not with the intensity at which the exercise was performed. However, these findings should be confirmed in future studies, because the results could have been masked by BMI, age, and physical activity level of the participants included in the different studies. This might be partially explained by a tendency to use walking as the exercise intervention if participants are overweight, elderly, or sedentary;^64,65^ and jogging if the subjects are not obese, younger, or physically active.^22^

Post meta-analytical studies assessing resistance training programs are needed, since reductions in dBP were found with a greater number of resistance exercises, although these exercises also led to a minor decrease in sBP. Because of the contradictory findings, it is likely that future studies may manipulate these variables to determine whether several resistance exercise sets reflect an increased workload and, therefore, a greater PEH,^63,66^ or if the design of the program should require several resting periods between exercises to dampen the BP elevation that normally occurs during resistance exercise^67^ in order to facilitate the onset of the PEH.

One implication arising from this meta-analysis affects the prescription of exercise. It is necessary to determine whether the PEH is greater as the exercise workload increases, ^63,68^ and whether it varies in females according to the menstrual cycle phase.^52^ Other questions that remain to be answered include the duration of the PEH when the individual is performing daily living activities (*i.e.*, outpatient phase),^5,10^ and what is the role played by genetics in triggering the PEH response.^69,70^

## Conclusion

In conclusion, regardless of the characteristics of the sample and exercise, the BP reduced in the hours following an acute exercise session. However, the reduction was greater if the exercise was performed as a preventive strategy and in physically active individuals who were not yet medicated.
